# Early Detection Biomarkers for Ovarian Cancer

**DOI:** 10.1155/2012/709049

**Published:** 2012-12-23

**Authors:** Sreeja Sarojini, Ayala Tamir, Heejin Lim, Shihong Li, Shifang Zhang, Andre Goy, Andrew Pecora, K. Stephen Suh

**Affiliations:** ^1^The Genomics and Biomarker Program, The John Theurer Cancer Center, Hackensack University Medical Center, Hackensack, NJ 07601, USA; ^2^Genewiz, Inc., South Plainfield, NJ 07080, USA; ^3^The John Theurer Cancer Center, Hackensack University Medical Center, Hackensack, NJ 07601, USA

## Abstract

Despite the widespread use of conventional and contemporary methods to detect ovarian cancer development, ovarian cancer remains a common and commonly fatal gynecological malignancy. The identification and validation of early detection biomarkers highly specific to ovarian cancer, which would permit development of minimally invasive screening methods for detecting early onset of the disease, are urgently needed. Current practices for early detection of ovarian cancer include transvaginal ultrasonography, biomarker analysis, or a combination of both. In this paper we review recent research on novel and robust biomarkers for early detection of ovarian cancer and provide specific details on their contributions to tumorigenesis. Promising biomarkers for early detection of ovarian cancer include KLK6/7, GSTT1, PRSS8, FOLR1, ALDH1, and miRNAs.

## 1. Introduction

Among gynecological malignancies, morbidity and mortality rates are higher among ovarian carcinomas because early detection is difficult due to the absence of recognizable physical symptoms and a lack of sensitive screening methods. In 2012, a total of 22,000 new cases and more than 15,000 deaths are expected, according to Cancer Facts and Figures, 2012, by the American cancer Society [1]. Despite availability of current screening measures, such as transvaginal ultrasound, measurement of biomarker CA125 levels [2], or a combination of both modalities, due to the highly heterogeneous nature of ovarian cancer mortality rates remain high. Although death rate has decreased by 1.9% every year from 2004 to 2008, ovarian cancer still accounts for 3% of all malignancies among women [1]. The long-term survival rate is less than 30% for advanced stage patients, but conventional surgery with chemotherapy can cure about 90% of patients if diagnosed in stage I. Indeed, if the malignancy arises in the ovary and is localized for a sufficient interval to permit effective screening, then the chances for survival are significantly higher [3]. Because their anatomical location is deep down the pelvis, tumor-related abnormal functioning of the ovaries is asymptomatic until the tumor becomes enlarged or disseminates. In postmenopausal women, the problem is exacerbated because ovaries become dysfunctional after menopause. Therefore, ovarian cancer is more likely to be detected in an advanced rather than an early stage [4]. Microarray analyses and proteomics have been promising technologies used in research to identify molecular signature biomarkers for early detection, disease classification, and prognosis of ovarian cancer. Collections of heterogeneous neoplasms comprising ovarian carcinomas have conventionally been classified based on their type and degree of differentiation. However, current clinical management practices overlook the heterogeneity of ovarian carcinoma [5]. Germline mutations in *BRCA1* and *BRCA2* confer higher risk of ovarian cancer; the estimated risk for *BRCA1* mutation carriers range between 16% and 68% by age 70 and between 11% and 27% for *BRCA2 *mutation carriers [6–10]. If diagnosed at a localized stage, the 5 yr survival rate is 93%; however, only 15% of all cases are detected at this stage. The majority of cases (63%) are diagnosed after dissemination with the 1-, 5-, and 10-year relative survival rates being 75%, 44%, and 35%, respectively [1]. Clinical trials for identifying *BRCA1* and *BRCA2* mutations in high risk populations are currently being performed (Table 2). As described in earlier reviews, both cytoreductive surgery and combination chemotherapy with platinum-based compounds and taxanes did not change the overall cure rate of ovarian cancer; however, the 5 yr survival rate has increased from 37% (1974–1976) to 46% (1999–2005) [11]. In order to improve long-term survival of patients, to improve the clinical outcomes of ovarian cancer and to obtain significant reduction of risk, effective early detection methods using screening biomarkers with adequate sensitivity are urgently needed [12]. 

### 1.1. CA125 (Cancer Antigen 125)

**Figure 1:**
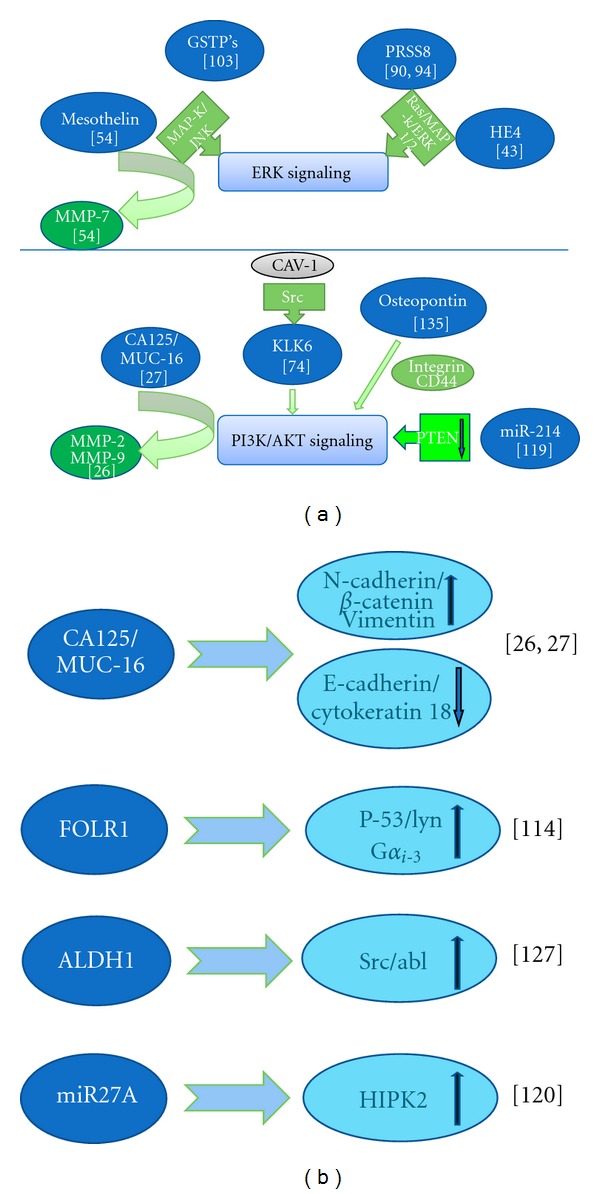
EGF/EGFR-based signaling pathways of ovarian cancer biomarkers. (a) Non-EGF/EGFR-based signaling pathways. (b) ↑ indicates upregulation. ↓ indicates downregulation. Caveolin-1 (Cav-1); phosphoinositide-3-kinase (PI3K); c-Jun N-terminal kinase (JNK); extracellular signal-regulated kinase (ERK); homeodomain-interacting protein kinase-2 (HIPK2); matrix metalloproteinase-2 or 7 (MMP-2 or MMP-7); multidrug-resistant protein (MDR1, P-glycoprotein); multidrug resistance-associated proteins 1 and 2 (MRP1/2); mitogen-activated protein kinase (MAP-K); phosphatase and tensin homolog (PTEN).

The widely used, classic, “gold standard” tumor biomarker, CA125, a high molecular weight glycoprotein, has a sensitivity between 50% and 60% with a specificity of 90% in early stage postmenopausal women, and expression of CA125 is enhanced in 90% of patients with epithelial ovarian cancer above normal levels [13–17]. CA125 is normally expressed in tissues derived from Mullerian and coelomic epithelia and is the only biomarker currently widely used in cancer therapy [18]. It was suggested that CA125 can potentially be used for early detection of ovarian cancer [19] since increased levels of CA125 may precede clinical detection by more than a year. In addition, analysis of CA125 levels has been useful in monitoring chemotherapy responses, distinguishing malignant pelvic masses from benign masses, detection of recurrence, and improving clinical trial design. A decline in expression of CA125 is considered a favorable prognostic occurrence during chemotherapy, and serial measurement of CA125 is used as an indicator of therapeutic outcomes and for assessing stabilization of the disease [15, 20, 21]. However, several factors undermine the significance of CA125 as an early detection biomarker. CA125 expression is absent in about 20% of ovarian cancers, and CA125 expression is elevated in some benign conditions such as liver cirrhosis, endometriosis, and peritonitis. Also, CA125 levels exhibit fluctuations associated with menstrual cycle and pregnancy. As a result, no CA125-based screening techniques are as yet recommended for the general population. However, CA125 has been used effectively in concert with other markers to increase its sensitivity as an early detection biomarker. In a study by Tcherkassova et al., the receptor for circulating fetal protein alpha-fetoprotein (RECAF), an oncofetal antigen, has been examined as a biomarker for early detection of ovarian cancer in conjunction with CA125 among healthy women. When specificity was set at 100% (for each of the individual markers), it was observed that the addition of RECAF to CA125 enhanced the sensitivity of detection to 83%, as compared to 70% when using CA125 alone. For stages III/IV the sensitivity increased from 79.6% to 88.2% with the addition of RECAF, and a more profound increase was observed for early detection of stages I/II (58.1% with CA125 alone to 76% with RECAF/CA125) [22]. Therefore, because of the relatively low sensitivity of CA125 as a single screening biomarker, combining it with additional biomarkers to create a multiple biomarker panel was more effective; no single biomarker can provide all the necessary information for ovarian cancer diagnosis and therapy. Currently, various clinical trials are evaluating CA125 alone or in combination with other biomarkers for screening of ovarian cancer (Table 2) [23–25]. It was demonstrated that CA125 binds to E-cadherin and *β*-catenin complexes, which results in enhanced motility, migration, and invasiveness of cells expressing CA125/MUC16 (Figures 1(a) and 1(b)) [26, 27]. As with some other ovarian cancer biomarkers, CA125/MUC16 expressing cells signaling enhance epidermal growth factor receptor (EGFR) activation, which results in increasing its downstream effectors Akt and ERK1/2 and in enhanced MMP-2 and MMP-9 expression [26]. Implementation of computer technology and statistical methods in developing better detection and treatment capacity of ovarian cancer has generated new tools that could boost sensitivity of CA125. One is a computerized algorithm which incorporates and stratifies an individual's age-specific risk for ovarian cancer using CA125 profile; risk of ovarian cancer algorithm (ROCA) increases the sensitivity of CA125 (86%) in preclinical detection. Using ROCA, it could be predicted whether or not an individual is at high risk based on the levels of CA125 (current and previous) as her age progresses, meaning if the levels of CA125 increase as the individual ages, ROCA identifies the individual as at high risk. Additionally, women with elevated levels of CA125 over 35 u/mL (which is considered as a threshold), which remain unchanged over the years, are identified as at lower risk (specificity 98%). Based on ROCA scores, women are thus triaged into low risk, high risk, and intermediate risk and referred for further procedures such as annuals, transvaginal sonography (TVS), or repeated evaluations of CA125 levels, respectively [28–30]. Similarly, Ova1 is an FDA approved multivariate index for identifying high risk ovarian tumors before any surgical procedures. It combines measurements of five proteins CA125-II, apolipoprotein A1, transthyretin, beta 2 microglobulin, and transferrin. Proprietary OvaCalc software is used to interpret the results, and an Ova1 score will be assigned which varies based on menopausal status. Ova1 score 5 and 4.4 is considered with higher risk of malignancy in premenopausal women and for postmenopausal, respectively, with sensitivity of 92.5% and specificity 42.8% in a trial conducted on women (*n* = 516) referred for surgery by physicians [31]. In a recent study involving 590 women with different types of malignancies including nonepithelial and epithelial ovarian cancers, malignancies metastatic to the ovary, other pelvic cancers, and borderline tumors, Ova1 demonstrated higher sensitivity compared to physician's assessment or to CA125 profile and identified the risk of malignancies when combined with physician assessment before surgery. However, this study demonstrated that Ova1 is independent of cancer stage and menopausal status of women and has high sensitivity in detecting ovarian cancer compared with CA125 and physician assessment [32]. It also demonstrated higher sensitivity in detecting ovarian cancer compared with CA125 alone. 

### 1.2. HE4 (Human Epididymis Protein 4)

HE4 is a member of the WFDC family of proteins (whey acidic four-disulfide core) and is found to be overexpressed in ovarian carcinomas. Normal functions of HE4 are yet to be identified; however, the specificity and sensitivity of HE4 shows promise as a serum marker for ovarian cancer in the early detection process [33, 34]. Currently, the FDA has approved the use of HE4 as a tumor marker for monitoring relapse or progression of EOC (epithelial ovarian carcinoma) [35]. Earlier studies evaluated HE4 alone and in combination with CA125 as a biomarker for ovarian cancer. The results suggested that HE4 used in conjunction with CA125 yielded significantly greater specificity than either markers alone [36]. Also, as a single marker, HE4 had the highest sensitivity (72.9% at 95% specificity), and when combined with CA125 sensitivity increased to 76.4% (at 95% specificity). Among biomarkers tested, HE4 levels demonstrated the highest sensitivity for stage I disease, but was only 45.9% at 95% specificity. There was no significant change in sensitivity for stage I disease when HE4 was combined with CA125 or with other biomarkers. Thus, HE4 complements the efficacy of CA125 in improving screening and diagnosis, and together they comprise a promising biomarker panel for detection and risk stratification of ovarian cancer [35, 37–40]. Recent research by Escudero et al. comparing tumor markers HE4 and CA125 in healthy individuals (*n* = 101), patients with nonmalignant lesions (*n* = 535), and patients with malignant tumors (*n* = 423) indicated that HE4 has higher specificity in patients with benign gynecological disorders than CA125. Similar results were obtained in patients with renal failure or disease. However, the levels of CA125 were higher in all nonovarian malignancies, and the results of this study suggest that even though HE4 has a higher diagnostic specificity than CA125, a combination of both improves the early detection and diagnosis of ovarian cancer of any histological type or stage [41]. In another study conducted among Chinese women (*n* = 491), analysis of HE4 and CA125 in sera from healthy subjects, patients with nonmalignant disorders and ovarian cancer patients showed that both CA125 and HE4 levels were elevated significantly in ovarian cancer patients compared to other groups, with the specificity of HE4 ranging from 90% to 100% and CA125 from 36% (benign gynecologic disease) to 99%, attaining a specificity of 100% for ovarian cancer with the combination of both biomarkers [37]. Furthermore, in a model proposed by Yurkovetsky et al. [42], a multibiomarker panel with CA125, HE4, CEA, and VCAM-1 was highly recommended for early detection of ovarian cancer with 86% sensitivity and 98% specificity. Overall, available data indicates that HE4 could be a novel biomarker for early detection of ovarian cancer in high risk populations, and a multibiomarker panel with CA125 would be promising in detection, diagnosis, and prognosis. HE4 was shown to induce tumor cell adhesion, migration, and growth through the EGFR-MAPK signaling pathway (Figure 1(a)) [43]. In a recent attempt to obtain a better detection tool, serum levels of HE4 and CA125 were incorporated with menopausal status leading to the development of ROMA (risk of ovarian malignancy algorithm) in detecting ovarian cancer from benign pelvic masses even in early stages. ROMA stratifies these patients as high risk groups or low risk, based on ROMA score (numerical) calculated from the predictive index [44]. Recent studies demonstrated that ROMA exhibits high diagnostic accuracy in predicting epithelial ovarian cancer from pelvic masses. However, further research is required for evaluating ROMA in early detection of ovarian cancer [45, 46].

### 1.3. Mesothelin

Several studies have demonstrated overexpression of mesothelin (a glycoprotein present on mesothelial cells lining the pleura, peritoneum, and pericardium) in most epithelial ovarian cancers and have suggested the eligibility of mesothelin as a target for cancer therapy [47, 48]. Previously Scholler et al. demonstrated that cancer cells undergo CA125/mesothelin dependent cell adhesion in the mesothelial epithelium of peritoneum and confirmed CA125 and mesothelin mediate cell attachment [49]. Rump et al. reported that, this mesothelin/CA125 interaction may also play a role in peritoneal metastasis of ovarian cancer [50]. In a recent study, Lowe et al. evaluated personal factors such as age, BMI, usage of talc, and smoking that influence the levels of expressions of mesothelin, CA125, and HE4 in high-risk, healthy postmenopausal women and demonstrated that “age” is a significant predictor in expression of mesothelin and HE4 since levels of these biomarkers were found to be increased in older women. Also, there was inverse correlation between mesothelin levels and BMI of the subjects (>50 yr *n* = 120, <50 yr *n* = 130) [51]. Similarly, a significant increase in levels of mesothelin in sera analyzed in normal subjects, subjects with benign disorders, and subjects with malignant ovarian tumors revealed that mesothelin could be a novel biomarker and that higher levels denote poor overall survival in patients following optimal debulking surgeryor who have advanced stage ovarian cancer [52]. Moreover, 42% of patients with early stage ovarian cancer had elevated mesothelin in urine compared to only 12% of patients who had elevated mesothelin in serum, suggesting the potential of mesothelin as an early detection biomarker [53]. Also, McIntosh et al. noted that mesothelin and CA125 as a combined marker provided greater sensitivity for early ovarian cancer diagnosis [19]. Cancer cells overexpressing mesothelin demonstrated enhanced migration and metastasis. These activities were mediated through MMP-7, which is regulated through the ERK1/2, Akt, and JNK pathways. The signaling pathway of mesothelin in ovarian cancer is detailed in Figure 1(a), [54].

### 1.4. Kallikreins

The human kallikrein (KLK) gene family, localized on chromosome 19q13.4, is composed of 15 genes encoding low molecular mass serine proteases (30 KD) of known or predicted trypsin-like or chymotrypsin-like activity, which dysregulate different types of cancer including ovarian, giving either a favorable or unfavorable prognosis [55–57]. KLKs are translated as preproenzymes and are cleaved into proenzymes upon release from the secretion pathway. Processing of the proenzymes into active extracellular KLKs is mediated by KLKs or other proteases [58, 59]. Despite the fact that KLKs are involved in the regulation of many physiological processes, including smooth muscle contractions, hormonal regulation, vascular cell growth/repair, and blood pressure, the role of KLKs in pathogenesis or progression of cancer and diabetes remains unclear. The role of KLKs in controlling cellular processes such as neovascularization, apoptosis, and tumor metastasis by cleavage of growth factors, extracellular matrix, or hormones has been previously reported, and robust arteriogenesis induced by overexpression of hK1 has been recently studied [60–62]. KLKs function in numerous physiological and pathological processes, including hormonal regulation [63], either individually or in pathways. Their genetic polymorphisms including sequence and splice variants are often associated with increased risk for various types of cancers including ovarian, thus revealing the potential role of KLKs as prognostic, diagnostic, and predictive biomarkers. KLK4-8, KLK10-11, and KLK13-15 were shown to be upregulated in ovarian tissue and serum from patients and were upregulated in cell lines at the mRNA and/or protein level. Previous studies reported that KLK4 (hK4) proteins are present in normal prostate tissue and are secreted in seminal plasma; however, higher levels of KLK4 expression are associated with the progression of ovarian cancer, mainly late stage serous epithelial-derived ovarian carcinomas where hK4 represents a potential biomarker for diagnosis and prognosis [64, 65]. 

KLK4 and KLK5 were reported to be associated with poor outcome in grade 1 and 2 tumors, indicating their association with aggressive forms of cancer. The association of KLK4 with aggressive cancer was identified in an RT-PCR study of KLK4 expression in 147 ovarian cancer tissue samples [66, 67]. Similar patterns of expression were observed in the levels of KLK5 with higher expression in aggressive serous carcinomas compared to expression in normal ovarian tissues or low grade tumors [68]. 

As demonstrated in Shan et al., KLK6 was reported to be a novel biomarker for ovarian cancer diagnosis based on the fact that it is associated with late stage,chemotherapy responsive, disease-free survival and serous histotype [69, 70]. KLK6 has been identified as having high potential as a novel biomarker with better specificity than CA125 for early detection of ovarian cancer because it is not elevated in noncancerous tumors [55]. Nonetheless, the diagnostic sensitivity is low compared to the diagnostic sensitivity of CA125. However, when KLK6 is used in combination with CA125, the sensitivity of each of the biomarkers is significantly increased (at 90% specificity, sensitivity is 72% for all patients and 42% in early stage patients) [55]. Using an immune-fluorometric assay KLK6 was found in high concentrations in various body fluids including CSF, breast milk, nipple aspirate fluid, and breast cyst fluid of women and in male and female serum [71, 72]. However, the sensitivity and specificity of both KLK6 and CA125 are ineffective in screening a population for early detection of ovarian cancer [73]. The prognosis for patients with preoperative KLK6 levels >4.4 *μ*g/L in serum is much worse than for patients with lower preoperative KLK6 serum levels. The significance of KLK6 as a prognostic factor is higher than CA125. The extensive and almost exclusive sialylation of KLK6 from malignant ovarian cells suggests that sialylated KLK6 could serve as a novel biomarker for early detection [74]. The signaling pathway of KLK6 in ovarian cancer is given in Figure 1(a), where its expression was found to be upregulated through downstream pathways of k-ras. A component of the plasma membrane Caveolae, CAV-1, was shown to be responsible for KLK6 gene expression and related protein secretion [75]. 

Another important kallikrein family member, KLK7, a chymotryptic serine protease previously reported to have a role in the desquamation of plantar stratum corneum, catalyzes the degradation of desmosomes in the deeper layers of skin during reconstruction [76], thus playing a pivotal role in cell shedding. Similarly, the presence of KLK7 on the surface of cancer cells suggests that, by digestion of extracellular matrix, KLK7 helps in the shedding of tumor cells and, therefore, in invasion and early metastasis. The significance of KLK7 in ovarian cancer early detection is directly related to its upregulated levels in ovarian cancer cells [77]. In a study of 44 ovarian tumors (12 low malignant and 32 carcinomas), Tanimoto et al. [78] showed that levels of KLK7 mRNA were elevated in 66.7% of low malignant potential tumor cells and in 78.1% of malignant cells, suggesting that the overexpression of KLK7 in ovarian tumors contributes to tumor cell growth and metastasis. 

KLK8 is normally expressed in ovaries as well as in adult and fetal kidneys, salivary gland, skin, tonsil, and breast. It is also detected in breast milk and amniotic fluid as well as in CVF, CSF, and ovarian cancer ascites [79]. 

Analysis of kallikreins 4–8, 10, 11, 13, and 14 levels in effusion supernatants obtained from 221 ovarian cancer samples and nonneoplastic diseases demonstrated that, with the exception of KLK4, all kallikreins were expressed at higher than normal levels in ovarian cancer effusions. Among these, KLK6, KLK7, KLK8, and KLK10 showed the highest statistical significance in ovarian cancer effusions over other cancer groups, suggesting that these kallikreins might be useful biomarkers in differential diagnosis of ovarian cancer [80]. In an analysis of kallikreins 6, 10, CA125, and hemostatic markers and 5-year survival outcome from epithelial ovarian carcinoma, it was found that ovarian carcinoma patients who lived past 60 months shared similar elevated preoperative levels of KLK10 and CA125 seen among benign cyst patients. However, the authors indicated a need for a further enlarged study to confirm these findings [81]. Results from an ovarian cancer xenograft model suggest that KLK10 has a tumor suppressive function [82]. Expression of KLK10 is noted in a variety of tissues, including breast, ovary, colon, prostrate, and testes [83, 84]. 

The observed upregulation of KLKs in ovarian cancer is important for diagnosis, prognosis, and treatment. KLK6, KLK10, and KLK11 may provide novel serological diagnostic markers since their expression levels in serum are significantly higher in ovarian cancer patients than in healthy subjects. Similarly, KLK4 and KLK9 share prognostic value in ovarian cancer, with higher expression of KLK5 correlating with poor prognosis [66]. In recent studies, we used a bioinformatics-guided approach coupled with subsequent screening and validation methods for identifying novel biomarkers for ovarian carcinoma. Our results showed that KLK6 and KLK7 are upregulated in ovarian cancer tissues over other cancer types. Upregulation occurs during early stages and in ovarian carcinomas of low malignancy, and these KLKs are secreted into the blood during tumor progression [85]. Hence, KLK6/7 could be further evaluated as early detection biomarkers.

### 1.5. PRSS8

Human prostasin (*PRSS8*), a trypsin-like proteinase (40 KDa) localized on chromosome 16p11.2, was first isolated from seminal fluid and was found to be localized or secreted (or both bound and secreted) on the apical surface of the epithelia of the lung, kidney, and prostate. Prostasin plays a significant role in activating epithelial sodium channels and suppressing the in vitro invasiveness of both prostate and breast cancers [86–88]. Similarly, epidermal tight junction formation and terminal differentiation are connected to the matriptase-prostasin proteolytic pathway [89]. Recent studies showed that EGFR (epidermal growth factor receptor) protein expression and EGF-induced phosphorylation of Erk1/2 (extra cellular signal regulated kinases) were found to be downregulated by prostasin expression in PC-3 prostate cancer cells. Given that prostasin functions in EGFR signal modulation, a recent study concluded that it was significant in the regulation of placental trophoblast cell proliferation via the EGFR-MAPK signaling pathway, since this cascade regulates placental cytotrophoblast proliferation [90].

 The potential of prostasin/PRSS8 as a novel biomarker for ovarian carcinoma was suggested by Mok et al. using microarray technology to identify upregulated genes for secretor proteins. The results demonstrated overexpression of PRSS8 in malignant ovarian epithelial cells and stroma compared to the normal ovarian tissue with sensitivity and specificity of 92% and 94%, respectively [91]. A significant decline in postoperative serum levels of PRSS8 was observed in a majority of cases. Similarly, Costa et al. demonstrated significantly higher over-expression of prostasin mRNA in fresh-frozen ovarian cancer tissues than in normal controls [92]. Previous studies to determine the function of Zinc-finger protein 217 (ZNF217) using Affymetrix Gene Chip analysis in the ovarian cancer cell line, HO-8910, with HG-U133 plus 2.0 arrays demonstrated that silencing of the *ZNF217* gene resulted in downregulation (approximately 8-fold) of 164 genes compared to normal cells. The same study also confirmed downregulation of PRSS8 after silencing *ZNF217* expression indicating the significance of *ZNF217 *as a key regulator [93] and suggesting PRSS8 as a potential biomarker in ovarian carcinomas. The signaling pathway of PRSS8 in ovarian cancer is detailed in Figure 1(a) [90, 94].

### 1.6. Glutathione S-Transferase Polymorphisms

Functional polymorphisms of members of the Glutathione S-transferase family (GSTM1, GSTT1, and GSTP1) are the result of large deletions present in the structural gene, which in turn affect drug metabolism and influence the effects of chemotherapy in cancer patients. Allelic variants of GSTs catalyze the conjugation of glutathione to xenobiotic or endogenous substrates, including potentially toxic chemical compounds, and promote detoxification. Given that GST polymorphisms are highly expressed in the human ovary [95] and that polymorphisms of drug metabolizing enzymes influence the susceptibility to different types of cancer, studies on the role of GST polymorphisms in the response to chemotherapy in ovarian cancer therapy would be appropriate. Earlier epidemiologic studies did not confirm the association of GST polymorphisms with epithelial ovarian cancer [96], although they suggested that individuals with homozygous deletions of GSTM or GSTT have reduced or no GST activity, making elimination of electrophilic carcinogens difficult. In a study conducted by Beeghly et al. using DNA extracts from 215 primary epithelialovariancancer tissues, GSTT1, GSTM1, and GSTP1 genotypes were identified and assessed by multiplex PCR and PCR-RFLP. The study incorporated Cox proportional hazards regression to determine the association between GST polymorphisms and cancer progression. The results indicated that although none of the individual GST polymorphisms were associated with disease characteristics, when adjusted for disease stage or limited to late-stage patients, GSTM1 polymorphism conferred a better survival. More significantly, combination of no GSTM1 and low GSTP1 resulted in over 60% better progression-free survival and nearly 40% improved overall survival. Therefore, functional polymorphisms of GSTM1 and GSTP1 have important roles in survival of the patients [97]. Similarly, a meta-analysis, by Economopoulos et al. examining the association of GST polymorphisms and ovarian cancer risk, suggested that GSTT1, GSTM1, and GSTP1 polymorphisms did not seem to contribute any increased risk in individuals. The study included 2357 cases and 3044 controls (8 studies) of GSTM1 null polymorphism, 1923 cases and 2759 controls (6 studies) of GSTT1 null polymorphism, and 3 studies of GSTP1 Ile105Val. Because the populations studied were largely white, the authors indicated that the results could not be extrapolated to other populations, and further race-specific analyses were needed [98]. The role of GSTs is highly relevant in drug-resistant tumors where higher expression of GSTs could alter regulation of the kinase cascade during drug therapy [99]. Similarly, the imbalance between GSH and related enzymes could lead to various pathologies, including cancer, with the genetic polymorphisms of GST affecting susceptibility and progression [99]. Significant reduction in enzymatic activities and higher risk for malignancies are observed in homozygous “null” genotypes (deletion of GSTT1 or GSTM1 genes), because the detoxifying abilities of these individuals are low [100–102]. In ovarian cancer patients with a “double null” genotype, the observed prognosis was poor, along with diminished response to chemotherapy; however, patients with null genotypes for either GSTT1 or GSTM1 exhibited an increased survival rate after chemotherapy for invasive ovarian carcinoma [100–102]. Considering these results, it might be predicted that polymorphisms of GST (GSTT1 or GSTM1) could provide a novel biomarker for early detection and diagnosis of ovarian cancer, although further research is necessary. The signaling pathway of GSTPs in cancer is given in Figure 1(a), [103]. Although it is not yet clear what are the signaling pathways of the different subtypes of GSTP, it was suggested they may operate through the ERK pathway. 

### 1.7. FOLR1

FOLR1 (folate receptor alpha) is a membrane-bound receptor protein involved in transport of folate into cells and other cellular processes. Over-expression of FOLR1 was observed in 69% of uterine serous carcinoma [104]. Rapidly dividing cancer cells have an increased requirement for folate to maintain DNA synthesis, and as reviewed by Kelemen, the expression of FOLR1 is regulated by depletion of extracellular folate levels, accumulation of homocysteine, steroid hormone levels, genetic mutations, and certain transcription factors and cytosolic proteins [105]. Kelemen discusses the significance of folate levels in tumor etiology and progression, with suggestions for future research in FOLR1 gene expression and regulation [105]. Similarly, the over-expression of FOLR1 in various nonmucinous tumors of epithelial origin, includingovarian carcinoma, has been reported; however, its evaluation as a novel biomarker for early detection has yet to be confirmed. FOLR1 over-expression was confirmed in serous ovarian carcinoma in previous studies detailing clinicopathologic features and outcomes, as well as the relationship between FOLR1 and chemoresistance [106]. This study evaluated 91 specimens of serous ovarian carcinomas, and the results showed that over-expression of FOLR1 is a poor prognostic factor for disease-free survival and has a negative impact on overall survival of patients. Moreover, FOLR1 regulated the expression of bcl-2 and Bax and inhibited cytotoxic drug-induced apoptosis in in vitro apoptosis experiments. The results further support that FOLR1 could be a potential biomarker in detection, prognosis, and assessing chemotherapy responses of ovarian carcinoma [106]. In a recent study by van Dam et al., expression of folate receptor-alpha was further examined by using a detecting imaging agent, and intraoperative use of a folate-targeted fluorescence agent with fluorescence microscopy showed a strong signal for all folate-positive malignant tumors and no signal for all folate-negative malignant tumors or benign lesions [107].

 Similarly, analysis of the diagnostic and prognostic role of FOLR1 and FOLR3 in effusion cytology of ovarian cancer (*n* = 71), breast cancer (*n* = 10), and malignant mesothelioma (*n* = 10) using quantitative PCR and flow cytometry showed significantly higher concentrations of FOLR1 and FOLR3 in ovarian carcinoma samples compared to breast or mesothelioma. Furthermore, the high expression of folate receptors in ovarian carcinomas shown in this study supports the validity of FOLR1 as drug targets in chemotherapy of ovarian cancer, since FOLR1 expression effectively differentiates ovarian cancer tumors with its coexpression with FOLR3, affecting the serosal cavities of tumors [108]. An earlier study to evaluate the significance of expression of folate receptors in gynecologic tissues (ovary, uterus, and cervix) by Wu et al. revealed contrasting expression patterns of FOLR1 between normal differentiation and malignant transformations of these tissues using quantitative analysis of FOLR1 mRNA. Results indicated that in normal ovary, FOLR1 expression was limited to germinal epithelium, and downregulation of FOLR1 was noted in differentiation of these cells into benign mucinous or benign serous lesions. Similarly, malignant transformation of these cells also resulted in down regulation of FOLR1, with higher levels of mRNA expression in serous cystadenocarcinoma [109]. Consequently, these studies support the upregulation of FOLR1 in ovarian cancer and confirm that it plays a significant role in regulating folate pathways in the tumor environment, making FOLR1 a possible biomarker for early detection of ovarian carcinoma. Clinical trials are currently being performed to evaluate the potential of FOLR1 as an early detection biomarker (Table 2). The difference in levels of expression of FOLR1 reported in recent studies is summarized in Table 4 [110–113]. Nearly no information is available in regard to the signaling pathway of FOLR1 in ovarian cancer, but it was suggested it signals through p-53/lyn/G*α*_*i*-3_ (Figure 1(b)) [114].

### 1.8. miRNA

In addition to the above-mentioned biomarkers, epigenetic markers including microRNAs (miRNA) are being considered as positive predictive biomarkers for the clinical management of ovarian cancer [115]. Carcinogenesis is a multistep process involving genetic alterations in oncogenes such as deletions, mutations, or amplifications and changes in microRNA genes. Iorio et al. investigated the importance of miRNA in ovarian cancer and demonstrated that miR-21, miR-141, miR-200a, miR-200c, miR-200b, miR-203, miR-205, and miR-214 could be used as diagnostic markers in ovarian cancer [116]. Taylor et al. compared these miRNA profiles in circulating tumor exosomes isolated from sera of both benign and malignant ovarian carcinoma patients. The results showed miRNA profiles in exosomal microRNA from ovarian cancer patients were significantly different from the profiles observed in patients with non-malignant disorders, with no exosomal miRNA detected in normal controls [117]. These results suggest that miRNA profiling could be a promising biomarker for early detection of ovarian cancer and biopsy profiling, as well as for screening asymptomatic populations. Further research in OVCAR3 cell lines showed higher levels of miR-21, miR-203, and miR-205 in ovarian cancer compared to normal ovary. miRNA levels were further increased when OVCAR3 cell lines were demethylated with 5-aza-2′-deoxycytidine, suggesting DNA hypomethylation as a possible reason for over-expression of miRNA. This study indicates the pathogenetic role of miRNA in epithelial ovarian cancer and supports miRNA gene methylation as a possible epigenetic pathway for their abnormal expression [116]. In addition, the role of miRNAs in disease prognosis and prediction of outcome in ovarian cancer has also been investigated by profiling miRNA expression from advanced cancer samples [118]. The results indicated that miR-200a, miR-200b, and miR-429 play a role in cancer recurrence and overall survival and demonstrated that low expression of miRNA 200 miRNAs in this group predicts poor outcome, whereas high expression of miRNA 200 miRNAs inhibits ovarian cancer cell migration, possibly preventing metastasis, which might indicate a better outcome [118]. The results discussed above indicate that miRNAs are aberrantly expressed in ovarian carcinoma and are potential biomarkers for early detection, diagnosis, and monitoring the overall progress of the disease. miR-214 was shown to operate through PI3K/AKT upregulation via PTEN suppression, while it was suggested that miR-27A in ovarian cancer signals through HIPK2 (Figures 1(a) and 1(b)) [119, 120].

### 1.9. ALDH1 (Aldehyde Dehydrogenase 1)

Being a member of aldehyde dehydrogenases protein family, ALDH1A1 plays important role when expressed in a subpopulation of cells with tumor-initiating properties in a variety of malignancies and thus a possible candidate biomarker in cancer therapy. ALDH1 is encoded by ALDH1A1 gene located in chromosome 9q21 and plays key role in pyridine nucleotide-dependentoxidation of aldehydes to respective carboxylic acids. The role of ALDH1 in differentiation of ovarian cancer stem cells and association of ALDH1 expression and various clinicopathologic factors including diagnosis, tumor grade, chemoresponses, staging of disease, and overall survival and disease-free survival of ovarian cancer was evaluated in recent research by Chang et al., using microarray analysis of ALDH1 (*n* = 442) by immune-histochemical staining as compared to the variations in clinical outcome. Results demonstrated that ALDH1 expression was associated with longer overall survival of the patients, and high expression of ALDH1 is a favorable prognostic factor in patients with ovarian cancer [121]. Similarly, recent study evaluating the expression of ALDH1 in epithelial ovarian cancer stem cells by Steffensen et al. demonstrated the higher expression of ALDH1 in CD44^+^ EOC stem cell clones [122] indicating ALDH1 as a potential biomarker for identifying presence of tumorigenic stem cells and improved therapy options. 

 Furthermore, tumorigenicity of stem cells coexpressing ALDH1 and CD 133 was studied by Silva et al., who demonstrated that tumor cells coexpressing ALDH1 and CD 133 have highly aggressive phenotype, rapid tumor formation and propagation, worse progression free survival and overall survival in ovarian cancer [123]. Considering these results, which demonstrate that ALDH1A1-positive ovarian cancer cells have increased tumorigenicity and higher chemoresistance, it might be predicted that ALDH1A1, particularly in a marker set, could be a possible biomarker for early detection of ovarian carcinomas [111]. Recently reported levels of expression of ALDHA1 in various ovarian cancers are detailed in Table 3 [121, 124–126]. The signaling pathway of ALDH1 in ovarian cancer is shown in Figure 1(b) [127].

### 1.10. Other Relevant Biomarkers

Multianalyte-based analytical discovery platforms readily adaptable to clinical diagnostic screening tests are used currently to profile immune responses against tumor-associated antigens. A goal is to identify tumor-specific antibodies present before the development of clinical symptoms that have potential for detecting ovarian cancer. Such antitumor immune responses are highly beneficial in identifying ovarian cancer [128]. Similarly, tumor vasculature also expresses significant differences from its normal counterpart and is a source of unique markers for detecting various malignancies including ovarian cancer. By using immunohistochemistry-guided laser-capture microdissection and genomewide transcriptional profiling for evaluating the differential expression of genes between tumor cells and normal ovarian tissues, studies have revealed the potential of TVMs (tumor vascular markers) as early detection biomarkers for ovarian cancer [129, 130]. Another potent early detection biomarkers for ovarian cancer are glycans and their associated proteins and lipid structures, which also vary between normal tissue and malignant tumors. Glycosylation is a complex posttranslational modification, and monitoring glycosylation changes provide a more specific and sensitive method for identifying malignancies including ovarian cancer [131, 132]. Microvesicles or exosomes are membranous bodies released from tumor cells and contain macromolecules including RNA, proteins, and lipids. Current research is focusing on identifying tumor exosomes as novel biomarkers for tumor environments since tumor exosomes act as central mediators expressing molecules involved in angiogenesis, stromal remodeling, chemoresistance, activating signaling pathways, and intercellular genetic exchanges [133]. Similarly, the efficiency of FDG-PET/CT (F-18 fluorodeoxyglucose-positron emission tomography) to visualize the increased glucose consumption of malignant lesions, especially in ovarian cancer, is discussed by Nowosinska et al. In that study, primary malignant tumors could be detected with more accuracy than borderline ovarian tumors; however, limitations included the inability to differentiate between benign and malignant pelvic masses. This may be developed into a potential technique for early detection of ovarian carcinoma and may have application in management of patients [134]. In addition, osteopontin (Figure 1(a)) [135] and B7-H4 have recently been identified as early detection biomarkers for ovarian cancer. These markers are undergoing further research for confirmation [136–138] (Table 1). A recent cell culture study revealed that geometric mean ofexpression levels of osteopontin in epithelial ovarian cancer cell lines is significantly higher (270.4) than healthy ovarian epithelial cell lines (4.1). Similarly, tissue level expression of osteopontin also varied from normal ovarian epithelial tissue (9) to epithelial ovarian cancer tissue (164). Moreover, immune localization of osteopontin showed higher levels of expression in borderline tumors than benign tumors, suggesting the importance of osteopontin as an early detection biomarker for ovarian cancer [138]. YKL-40, a glycoprotein in chitinase protein family, expresses elevated levels in early and advanced stages of ovarian cancer. Serum levels of YKL-40 from normal healthy individuals, patients at high risk for developing ovarian cancer, and ovarian cancer patients were assessed in a study by Dupont et al. which demonstrated that higher levels of YKL-40 were observed in stage I and stage II patients. Furthermore, YKL-40 levels reliably predicted recurrent and advanced ovarian cancer in these study cohorts since increased levels were observed during advancement of disease [139] indicating that YKL-40 may represent a potential biomarker for early detection of ovarian cancer.

### 1.11. Genetic Biomarkers

Ovarian cancer, as any other cancers, arises from cells that acquire and accumulate DNA sequence variations. Some of those sequence variations confer the cells a growth advantage and lead to their uncontrolled proliferation (tumorigenesis), unchecked migration (metastasis), and survival against various odds (drug resistance). The advance of sequencing technology is making it possible to uncover those genetic drivers and, thus, identify genetic biomarkers to aid early detection, disease subtyping, staging, and prediction of disease prognosis and selection of effective therapy. The advancement in isolation of small number of circulating tumor cells will eventually make it possible to examine those genetic biomarkers early noninvasively.

As the tip of iceberg, mutations in multiple genes involved in DNA damage repair, cell cycles, cell metabolism, cell adhesions, and other pathways have been reported in association with ovarian cancer. For example, germline mutations in *BRCA1, BRCA2*, and *Rad51D* are well known to increase ovarian cancer risk [142, 143]. Whole exome sequencing of 489 high grade serous ovarian cancers (stage II to IV) confirmed the involvement of *BRCA1* and *BRCA2*, with 8%-9% of tumor containing germline mutation and 3% more containing somatic mutation in *BRCA1* and *BRCA2.* The study further identified other recurrently mutated genes, including *TP53, RB1, NF1, FAT3, CS*∖*MD3, GABRA6,* and *CDK12. *Specifically, 96% of the 489 samples contain mutations in *TP53* [144]. While *TP53* mutations are prevalent in high grade serous cancers, *KRAS* and *BRAF* mutations are more frequent in low-grade subtypes [145]. *CTNNB1* (beta-catenin) mutations are common in endometrioid carcinomas, *PICK3CA* mutations are most frequent in clear cell carcinoma, and *ARID1A* (the AT-rich interactive domain 1A) mutations are often observed in both tumor types [146–148]. We have taken advantage of semiconductor sequencing technology, prepared DNA from 22 serous and endometrioid tumor samples (1 FFPE slide per patient), and sequenced 64 selected genes. With several thousandfold of coverage, we have identified 9 other gene variants that occur in 62%–94% of patients (Li and Suh, *unpublished data*).

 Whole transcriptome and exome sequencing revealed that *DICER1* mutations occur at high frequency in nonepithelial ovarian cancers [149]. The mutations are clustered at the metal binding site of the RNase IIIb domain, which are critical for miRNA processing. As reviewed above in section, miRNA themselves are increasingly being considered as biomarkers for ovarian cancer development. 

## 2. Summary

Despite all the conventional and current methods used to detect ovarian cancer development, such as radiographic imaging, invasive biopsies, tumor markers, and a combination of transvaginal ultrasounds with tumor markers, ovarian cancer remains the most common gynecological malignancy and has the highest mortality rate. The identification and validation of early detection biomarkers highly specific to ovarian cancer are needed to establish minimally invasive screening methods for detecting early onset of ovarian cancer. Evaluation of promising biomarkers for early detection opens new horizons in ovarian cancer detection and therapy [150]. The analysis of the human serum proteome has provided better biomarker candidates for early detection, an important goal, as early diagnosis improves the five-year survival rate over 90%. We discuss CA125, a tumor marker with high discriminative power even before the onset of symptoms, which has been demonstrated in many ovarian cancer studies especially in postmenopausal women. We note, however, that the increase in levels of CA125 in other types of cancer, endometriosis, ovulation, other benign ovarian diseases, as well as its low sensitivity in early stages, limits its potential as a single biomarker for ovarian cancer screening. Consequently, a multibiomarker panel aimed at augmenting the sensitivity and specificity of CA125, in which CA125 is used with HE4, mesothelin (Table 1) [141], CEA, VCAM-1, B7-H4, YKL-40, or different combinations is under study for early detection. Of these, HE4 and mesothelin are the most promising candidates to date. Additionally, screening for germline mutations in *BRCA1/BRCA2* is also a promising method for early detection of ovarian cancer in current clinical practice since high risk populations with corresponding mutations could be genetically predisposed toward developing cancer. Prostasin (PRSS8), GSTT1, FOLR1, KLK6, KLK7, and ALDH1 are all currently under research and clinical trials (Table 2) and are also potential biomarkers for early detection of ovarian cancer. Mok et al. demonstrated the over-expression of PRSS8 in malignant ovarian epithelial cells and stroma with sensitivity and specificity of 92% and 94%, respectively, and with a significant decline in serum postoperative levels. Similarly, evaluation of GST functional polymorphisms (GSTT1, GSTM1) might help in detecting ovarian cancer at early stages, since they affect susceptibility and progression of cancer. However, additional research is needed for confirmation of these possibilities. Also, considering the fact that carriers of low function GST genotypes (GSTT1 null, GSTM1 null) have a strong survival benefit, evaluation of GST polymorphisms could be promising biomarker for early detection of ovarian cancer. Similarly, over-expression of folate receptor-alpha (FOLR1) in 90%–95% nonmucinous tumors of epithelial origin, including epithelial ovarian carcinoma (90%–95%) and serous tumors, indicates the possibility of FOLR1 as an early detection biomarker and suggests the need for further research for confirmation. Several studies demonstrate the significance of KLK6 and KLK7 in ovarian cancer, both being highly expressed in ovarian malignant tumors from early to advanced stages; however, the levels of these proteins in serum samples analyzed at Hackensack University Medical Center had the opposite signature, showing peaks in stage I which declined toward advanced stages [85]. These data support the classification of KLK6/7 as early detection biomarkers. Similarly, small noncoding microRNAs acting as epigenetic regulators cause post transcriptional silencing of target genes and inhibit the activity of antioncogenic pathways promoting tumorigenesis; the aberrant expression of miRNAs has been demonstrated in several studies. Häusler et al. demonstrated higher expression of miR-21, miR-141, miR-200a, miR-200c, miR-200b, miR-203, miR-205, and miR-214 and showed similarity in miRNA profiling in exosomal microRNA from ovarian cancer patients, suggesting that miRNA profiling could be a promising biomarker for early detection of ovarian cancer, biopsy profiling, and for screening asymptomatic populations (Table 1) [140]. Studies have demonstrated that ALDH1-positive ovarian cancer cells have increased tumorigenicity and higher chemoresistance; therefore, it could be predicted that ALDH1, particularly in a marker set, could be a possible biomarker for early detection of ovarian carcinomas (Table 3). 

In conclusion, the identification of novel and robust biomarkers with higher specificity and sensitivity for early detection of ovarian cancer could significantly improve the overall survival rate of ovarian cancer patients. The promising biomarkers in this category include KLK6/7, GSTT1, FOLR1, ALDH1, and miRNAs, along with multibiomarker panels in combination with CA125, which is widely used in current practice.

## Figures and Tables

**Table 1:** Specificity and sensitivity of early detection biomarkers for ovarian cancer from various studies.

Biomarker	Early detection biomarkers: ovarian cancer (sensitivity and specificity)								
	Source	*n* (Total)	Specificity	Sensitivity	Levels	Benign (*n*)	Other malignancies (*n*)	Ovarian (*n*)	Reference
HE4	Serum	233	95%	73%	High	166	NA	67	[39]
HE4 + CA125	Serum	472	74.20%	100%	High	383	89	10%	[36]
			76%	92.30%					
Prostasin + CA125	Serum	137	94%	92%	High	100	37	37	[91]
Osteopontin	Plasma	251	NA	NA	High	107	47	51	[138]
KLK6 (hK6)	Serum	384	95%	21%–26%	High	141	NA	146	[55]
KLK6 + CA125	Serum	384	90%	42%	High	141	NA	146	[55]
B7-H4	Serum	2256	97%	45%	High	1023	997	236	[140]
B7-H4 + CA125	Serum	2256	97%	65%	High	1023	410	236	[141]

*n*: number of patients.

**Table 2:** Clinical trials (currently active or completed) for evaluating novel biomarkers of ovarian cancer.

Biomarker	Clinical trials for evaluating early detection biomarkers in ovarian cancer (USA)						
	Condition	Phase	*n *	Status	Clinical trial no.	Reference	Primary outcome measure
All biomarkers	Adnexal mass	1	500 (E)	Not yet recruiting	NCT01466049	NA	Screening
HE4 + CA125	Pelvic mass	0	566	Completed	NCT00315692	[23]	cancer versus benign disease
CA125	Low risk (w)	1	9500 (E)	Recruiting	NCT00539162	NA	Rate of increase in CA125 over time
HE4 + CA125	Adnexal mass	1	512	Completed	NCT00987649	NA	Initial cancer risk assessment
CA125 + HE4	High risk (w)	1	1208 (E)	Recruiting	NCT01121640	NA	PPV of screening protocols
CA125	High risk (w)	2	2400 (E)	Unknown	NCT00080639	NA	Screening
Mesothelin	Low risk (w)	0	250 (E)	Unknown	NCT000155740	NA	Screening
FOLR1	Stage I Ov ca.	2	50 (E)	Recruiting	NCT01511055	NA	Sensitivity and specificity of IOI with folate
CA125 + TVU	Ovarian diseases	0	750 (E)	Recruiting	NCT01292733	NA	CA125 measurement in blood over time
CA125 ± TVU	Postmenopausal	0	48230	Completed	NCT00058032	[24, 25]	Screening postmenopausal women
CA125	High risk (w)	0	2430	Recruiting	NCT00039559	NA	Feasibility at study completion
CA125 + TVU	High genetic risk (w)	0	5000 (E)	Unknown	NCT00033488	NA	Annual screening
CA125	High risk (w)	0	6000 (E)	Recruiting	NCT00005095	NA	Screening
Combined methods	Ov. neoplasms	0	36000	Not yet recruiting	NCT01178736	NA	Low-cost screening
Interventional	High risk (w)	0	1500	Recruiting	NCT00849199	NA	Genetic testing, screening
All Biomarkers	High risk (w)	0	250 (E)	Recruiting	NCT00854399	NA	Overall survival
Tumor markers	High risk (w)	0	5000	Completed	NCT00267072	NA	Early stage detection
DNA markers	Ovarian cancer	0	170 (E)	Recruiting	NCT00879840	NA	Assessment of screening modalities
BRCA 1/2 mutation	Ov. neoplasms	0	1500	Completed	NCT00001468	NA	Identifying BRCA 1/2 mutation

TVU: transvaginal ultrasonography. (w): women, (E): estimated enrollment, IOI: intraoperative imaging. Source: http://clinicaltrials.gov/.

**Table 3:** Levels of expression of biomarker ALDH1 in various stages of ovarian cancer.

Biomarker	Expression pattern on tumors	Category	*N *	Positive rates (levels of expression)	References
	Low to high	Serous stages III-IV	65	0% in 27.1% of samples	[124]
				1%–20% in 44% of samples	
				20%–100% in 28.9% of samples (10% of all patients demonstrated nearly 100% expression)	
	Low	Malignant tumors	5	17.1% ± 7.61%	[125]
ALDH1		Benign tumors	5	31.03% ± 6.68%	
		Healthy controls	5	37.4% ± 5.4%	
	Low to high	Serous carcinoma	266	>20% expression in 85% of samples	[121]
		Stage I	32	>20% expression in 44% of samples	
	Low and high	Late stage	65	77% positive cells	[126]

**Table 4:** Levels of expression of biomarker FOLR1 in various stages of ovarian cancer.

Biomarker	Expression pattern	Category	*N *	Positive rates (levels of expression)	References
FOLR1	High	Early stage (I/II)	15	16 ± 2 au	[110]
		Advanced stage (III/IV)	15	12 ± 2 au	
		Healthy controls	30	7 ± 0.9 au	
	High	Advanced stage	104	97%	[111]
		Healthy controls	30	Negligible	
	High	Primary tumors	186	72%	[112]
	Weak to moderate	Recurrent tumors	27	81.5%	[113]
		Serous carcinoma	210	81.8%	
		Nonserous carcinoma	116	39.9%	
